# Kinetic Parameters and Cytotoxic Activity of Recombinant Methionine γ-Lyase from Clostridium tetani, Clostridium sporogenes, Porphyromonas gingivalis and Citrobacter freundii

**Published:** 2013

**Authors:** E. A. Morozova, V. V. Kulikova, D. V. Yashin, N. V. Anufrieva, N. Y. Anisimova, S. V. Revtovich, M. I. Kotlov, Y. F. Belyi, V. S. Pokrovsky, T. V. Demidkina

**Affiliations:** Engelhardt Institute of Molecular Biology, Russian Academy of Sciences, Vavilova Str., 32, Moscow, Russia, 119991; Institute of Gene Biology, Russian Academy of Sciences, Vavilova St., 34/5, Moscow, Russia, 119334; Blokhin Cancer Research Center, Russian Academy of Medical Sciences, Kashirskoe sh., 24, Moscow, Russia, 115478; Gamaleya Research Institute of Epidemiology and Microbiology, Gamaleya Str., 18, Moscow, Russia, 123098

**Keywords:** kinetic parameters, methionine γ-lyase, pathogenic microorganisms, oligomeric structure, cytotoxicity

## Abstract

The steady-state kinetic parameters of pyridoxal 5’-phosphate-dependent recombinant methionine γ -lyase from three pathogenic bacteria, *Clostridium tetani*, *Clostridium sporogenes, *and *Porphyromonas gingivalis,* were determined in β- and γ-elimination reactions. The enzyme from *C. sporogenes *is characterized by the highest catalytic efficiency in the γ-elimination reaction of *L*-methionine. It was demonstrated that the enzyme from these three sources exists as a tetramer. The N-terminal poly-histidine fragment of three recombinant enzymes influences their catalytic activity and facilitates the aggregation of monomers to yield dimeric forms under denaturing conditions. The cytotoxicity of methionine γ-lyase from *C. sporogenes* and *C. tetani *in comparison with *Citrobacter freundii *was evaluated using K562, PC-3, LnCap, MCF7, SKOV-3, and L5178y tumor cell lines. K562 (IC_50_=0.4–1.3 U/ml), PC-3 (IC_50_=0.1–0.4 U/ml), and MCF7 (IC_50_=0.04–3.2 U/ml) turned out to be the most sensitive cell lines.

## INTRODUCTION

Methionine γ-lyase (MGL) [EC 4.4.1.11] is a pyridoxal 5’-phosphate-dependent enzyme that catalyzes the γ-elimination reaction of *L*-methionine, yielding methyl mercaptan, α-ketobutyric acid, and ammonia:

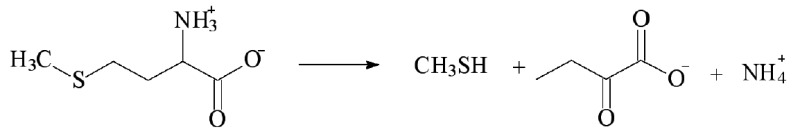



In addition to the physiological reaction, the enzyme catalyzes the β-elimination reaction of *L*-cysteine and its S-substituted derivatives, yielding the corresponding mercaptans, pyruvic acid, and ammonia [1]. The enzyme was isolated from *Pseudomonas putida*,* Aeromonas *sp., *Clostridium sporogenes*, *Porphyromonas gingivalis*, *Brevibacterium linens BL2*, *Citrobacter freundii*, and several others, from eukaryotic protozoa* Trichomonas vaginalis *and *Entamoeba histolytica*, and from fungi *Aspergillus flavipes *[2]. This enzyme is absent in mammalian cells but is present in pathogenic bacteria, such as *Aeromonas *sp. [3], *C. sporogenes *[4],* P. gingivalis *[5], and in pathogenic protozoa *E. histolytica* [6], *T. vaginalis *[7], which allows one to consider the enzyme as a potential target for novel antibiotics. The enzyme is of interest as an anticancer agent, since the growth of malignant cells of various origins (unlike the growth of normal cells) is accompanied by obligatory methionine utilization [8]. The possibility of development of an anticancer agent based on MGL from* P. putida *has been demonstrated *in vitro *and *in vivo* [9-12]. Antitumor activity of the enzyme from *A. flavipes* with respect to several human tumors was observed* in vivo *[13]. Several kinetic parameters of the recombinant MGL derived from the pathogenic microorganisms* C. tetani *(causative agent of tetanus), *C. sporogenes* (causative agent of gas gangrene and enteritis), and *P. gingivalis *(causative agent of periodontitis) were determined in the present study; the data on the cytotoxic activity of the enzyme from these sources and from *C. freundii *were analyzed.

## EXPERIMENTAL

**Bacterial cultivation and purification of the enzyme**

*Escherichia coli *BL21 (DE3) cells containing the MGL genes from *C. sporogenes*, *C. tetani*, and *P. gingivalis *in the plasmid pET -28a [14] were cultured in an “inducing” medium [15] at 37°C under stirring (180 rpm) for 24 h. The cells were harvested by centrifugation and stored at –80°C. Cell disruption and the removal of nucleic acids were performed according to [16]. After the separation of nucleic acids, the preparations were transferred to a 50 mM potassium phosphate buffer, pH 8.0, containing 0.05 mM pyridoxal 5’-phosphate (PLP) using a “Centricon-30 ultrafiltration unit” (Amicon, USA). The polypeptide chains of the enzymes isolated from three sources contained the poly-histidine fragment MGSSHHHHHHSSGLVPRGSH at their N-termini. The preparations were purified using affinity chromatography on a column with a Ni^2+^IMAC –Sepharose sorbent (GE Healthcare, Sweden); a 10–500 mM imidazole gradient in a 50 mM potassium phosphate buffer, pH 8.0, containing 0.05 mM PLP was used for MGL elution. Fractions with the characteristic spectra of pyridoxal 5’-phosphate-dependent enzymes with λ_max_ at 420 nm were obtained at an imidazole concentration of 25–155 mM. Cultivation of the biomass of *E. coli *BL21 (DE3) cells containing a plasmid with the MGL gene from *C. freundii *and purification of the enzyme was carried out as per [17]. The concentrations of the purified preparations were determined using the coefficient* А*_1%_^278^ = 0.8 [17]. The purity of the preparations was evaluated using electrophoresis under denaturing conditions according to the Laemmli method [18]. The bands in the electrophoregrams were identified by Coomassie R-250 staining [19] and Western blotting using the poly-histidine fragment HisProbe-HRP reagent (Thermo scientific, Rockford, IL, USA) [20]. The activity of the preparations in the γ-elimination reaction was determined by the rate of formation of α-ketobutyric acid in the coupled reaction with D-2- hydroxy isocaproate dehydrogenase under the conditions described in [17]. One unit of enzyme activity was defined as the quantity of MGL catalyzing the formation of 1.0 μM/min α-ketobutyrate at 30°C. The specific activities of the enzyme from *C. tetani*, *C. sporogenes*,* P. gingivalis *and *C. freundii *were 16.6, 12.8, 5.0, and 10.2 U/mg, respectively.

Cleavage of the His-tag fragment was performed in a reaction with thrombin. The reaction mixture (1 ml) containing 10 mg of the enzyme in a 50 mM potassium phosphate buffer, pH 8.0, 1 mM DTT , 0.05 mM PLP, and 100 units of thrombin were incubated for 24 h at 4°C. The product was then purified by gel filtration on a Superdex 200 column (GE Healthcare, Sweden) equilibrated with a 50-mM potassium phosphate buffer, pH 8.0, containing 1 mM DTT and 0.05 mM PLP. The homogeneity of the preparation was determined by electrophoresis under denaturing conditions.

**Determination of the oligomeric composition of MGL from *C. tetani*, *C. sporogenes*, and *P. gingivalis***

The molecular weights of the enzymes from *C. tetani*,* C. sporogenes*, and *P. gingivalis *were determined for the enzymes containing His-tag, and after the thrombin-facilitated cleavage of the tag, followed by gel filtration on a Superdex 200 10/300 GL column (GE Healthcare, Sweden). A 50 mM potassium phosphate buffer, pH 8.0, containing 0.05 mM PLP and 1 mM DTT was used for elution.

**Determination of the steady-state kinetic parameters of the γ- and β-elimination reactions**

The steady-state parameters of the γ-and β-elimination reactions were determined by the formation rate of α-ketobutyric and pyruvic acids in coupled reactions with D-2-hydroxy isocaproate dehydrogenase and lactate dehydrogenase under the conditions described in [17] by varying the substrate concentrations in the reaction mixtures. The data were processed according to the Michaelis–Menten equation using the Enzfitter software program. The molecular weights of the enzyme subunits with allowance for His-tag (which were equal to 44.04, 44.36, an 44.08 kDa for MGL from *C. tetani*,* C. sporogenes*, *P. gingivalis, *respectively) were used in the calculations.

**Evaluation of** *in vitro* **cytotoxicity**

The cytotoxic activity of MGL of various origins was evaluated for the Fisher L5178y lymphadenosis cell line (a collection of tumor strains from the N.N. Blokhin Cancer Research Center), PC-3 and LnCap human prostate cancer cell lines (ATCC , USA), the MCF7 human breast cancer cell line (ATCC , USA), K562 chronic erythroblastic human leukemia cell line (ATCC , USA), and the SKOV-3 human ovarian cancer cell line (a collection of tumor strains from the N.N. Blokhin Cancer Research Center). Cells were cultured at 37°C and 5% CO_2_ in the RPMI 1640 medium (PanEco, Russia) containing 10% of fetal bovine serum (HyClone Laboratories, UK), 2 mM *L*-glutamine, and 100 U/ml of penicillin and streptomycin (PanEco, Russia). The cells that reached the logarithmic growth phase were passed into 96-well flat bottom microplates (Costar, USA) – (4-6) X 10^4^ cells per well – and pre-incubated for 24 h prior to the addition of the test enzymes under the abovementioned conditions. Light microscopy of the cells was carried out using an AxioVision 4 system (Carl Zeiss, Germany). Cell viability was determined using trypan blue dye exclusion staining (PanEco, Russia). Cell count was determined in the Goryaev chamber.

The MGL preparations in the RPMI 1640 medium in a wide range of progressively decreasing concentrations were added in the wells with the cell culture and co-incubated for 72 h. In addition to MGL, the culture medium contained 5 X 10^-4^ M PLP. The range of enzyme concentrations in the culture medium corresponded to 0.000001–6.2 U/ml. An equal volume of the RPMI 1640 medium with PLP was added in the control wells. The level of cell metabolism following the incubation period was determined using a standard MTT colorimetric assay [21]. The optical absorption of the dimethyl sulfoxide colored solutions was measured using a Multiskan MS plan-table photometer (Labsystems, Finland) at λ = 540 nm. The viability of a cell culture after co-incubation with test substances was evaluated using the following formula: (*N*_о_/*N*_c_) X 100%, where *N*_o_ is the optical absorbance in the test samples and *N*_c_ is the optical absorbance in the control sample. The nonlinear regression method was used to calculate the inhibitory concentration of each enzyme in the medium; i.e., the concentration that caused a 50% reduction in the number of viable cells (IC_50_).

**Statistical data analysis**

The data were processed using the SPSS 11.5 software package. The relationships between IC_50_ and *K*_м_ were studied using the Pearson correlation analysis. The correlation coefficients were calculated for the grouped cytotoxicity data in various cell lines and for the ungrouped data. In the former case, the correlation analysis included the geometric mean of IC_50_ for various cell cultures. In the latter case, the cytotoxicity was individually assessed using each cell line; the data were pre-logarithmized for the symmetrization of the distribution law.

One-way ANOVA test was used to compare the cytotoxicity of the enzymes from *C. freundii*, *C. sporogenes*, and *C. tetani*. An analysis of the logarithmized data was conducted, since the dispersions of IC_50_ in the groups varied considerably. The mean values ± SD – arithmetic mean and standard deviation; geometric mean (antilog of the logarithmic means); *р*_ANOVA_= 0.005 – statistical significance of the differences according to the data from the analysis of the variance in general were calculated. The statistical significance of the differences in the cytotoxic activity of various enzymes was evaluated using the Tukey method.

## RESULTS AND DISCUSSION

**Kinetic parameters of MGL from three pathogenic bacteria**

We determined the steady-state kinetic parameters of MGL from three sources in the γ-elimination reaction for three substrates: *L*-methionine, *L*-methionine sulfoxide, S-ethyl-*L*-homocysteine, and in the β-elimination reaction for two substrates – S-ethyl-* L*-cysteine and S-benzyl-*L*-cysteine. The data are presented in Table 1 in comparison with the parameters for the MGL from *C. freundii*.

In general, the kinetic parameters for the three enzymes and MGL isolated from *C. freundii *were comparable. The enzyme from *C. sporogenes *is characterized by the highest catalytic efficiency in comparison with other enzymes (*к*_cat_/*К*_m_ value) in the reaction with the physiological substrate, and the enzyme from *P. gingivalis* is characterized by the lowest catalytic activity. As mentioned above, antitumor activity was previously determined primarily for MGL from *P. putida* [22, 23]. The *k*_cat_, *K*_m_, *k*_cat_/*K*_m_ values for the γ-elimination reaction of *L*-methionine for MGL from this source are equal to 25.39 s^–1^, 0.92 mmol, and 2.76 X 104 М^-1^s^-1^, respectively [24]; i.e., the enzyme from *C. sporogenes* is characterized by a higher affinity to *L*-methionine than MGL from *P. putida*, but their catalytic efficiency is virtually identical.

**Oligomeric structure of the recombinant proteins**

It has previously been demonstrated that MGL from* P. putida *exists in solutions as a tetramer [25]. The Xray diffraction analysis data for the recombinant MGL from *C. freundii *also indicated that the enzyme exists as a tetramer [26].

**Table 1:** Kinetic parameters of MGL from various sources*

Substrate	MGL from P. gingivalis			MGL from C. tetani			MGL from C. sporogenes			MGL from C. freundii**		
	k_cat_, s^-1^	К_m_, mM	k_cat_ /K_m_, M^-1^s^-1^	k_cat_, s^-1^	К_m_, mM	k_cat_ /K_m_, M^-1^s^-1^	k_cat_, s^-1^	К_m_, mM	k_cat_ /K_m_, M^-1^s^-1^	k_cat_, s^-1^	К_m_, mM	k_cat_ /K_m_, M^-1^s^-1^
L-Met	3.9	1.77	2.2×10^3^	12	0.947	1.27×10^4^	9.86	0.432	2.28×10^4^	6.2	0.7	8.85×10^3^
S-Et-L-Hcy	3.84	0.93	4.13×10^3^	5.89	0.545	1.08×10^4^	7.05	0.278	2.54×10^4^	6.78	0.54	1.26×10^4^
L-Met(SO)	5.05	12.22	4.13×10^2^	2.7	7.07	3.82×10^2^	6.7	33.51	2.0×10^2^	2.52	6.21	4.06×10^2^
S-Et-L-Cys	8.05	2.17	3.71×10^3^	7.08	0.72	9.83×10^3^	6.3	0.358	1.76×10^4^	5.03	0.17	2.96×10^4^
S-Bzl-L-Cys	5.8	1.47	3.94×10^3^	8.5	0.766	1.11×10^4^	10	0.348	2.87×10^4^	8.16	0.18	4.53×10^4^

* The mean squared error of the experiment in the determination of the kinetic parameters did not exceed 10%. ** Data from [16, 17].

**Fig. 1:**
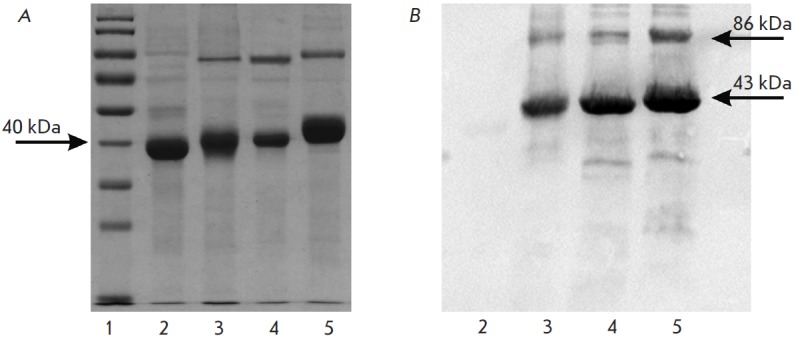
Electrophoresis (a) and western blot (b) of MGL from various sources. 1 – standard molecular weight markers, 2 – C. freundii MGL, 3 – P. gingivalisMGL, 4 – С. sporogenes MGL, 5 – C. tetani MGL

Two major bands have been revealed in the electrophoregrams of denatured preparations from *C. sporogenes*,* C. tetani*, and *P. gingivalis*. The first band with respect to the molecular weight corresponds to the MGL subunit; the second band is twice as large as the first one (*Fig. 1A*). Both of these bands interacted with the His-tag reagent (*Fig. 1B*). The bands corresponding to the dimeric form of MGL could form either during the oxidation of the sulfhydryl groups of MGL under the conditions of Laemmli electrophoresis, or their formation could be attributed to the His-tag. In most cases, the presence of the His-tag at the N-or C-terminus of the recombinant proteins did not affect their structure and function [27]; however, data on the influence of the His-tag on the structure and function of proteins are available. Thus, it has been demonstrated [28] that the presence of the His-tag at the C-terminus restores the ability of the mutant form of the DNA binding protein π^30.5^ to form dimers, as opposed to the wild-type protein that is incapable of dimerization. The presence of the His-tag at the N-terminus of galactitol-1-phosphate 5-dehydrogenase reduced the enzyme’s stability and resulted in aggregation of dimeric molecules [29].

It can be presumed that the presence of the MGSSHHHHHHSSGLVPRGSH sequence at the N-termini of the polypeptide chains of MGL from *C. sporogenes*,* C. tetani *and *P. gingivalis *affects the oligomeric organization of the enzymes.

**Fig. 2:**
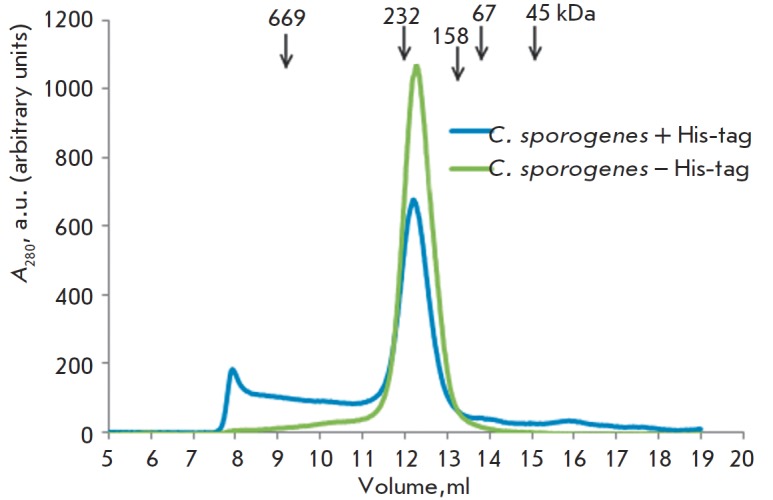
Gel filtration of С. sporogenes MGL. The column was calibrated using standard markers: their molecular weights are shown in the figure

**Fig. 3:**
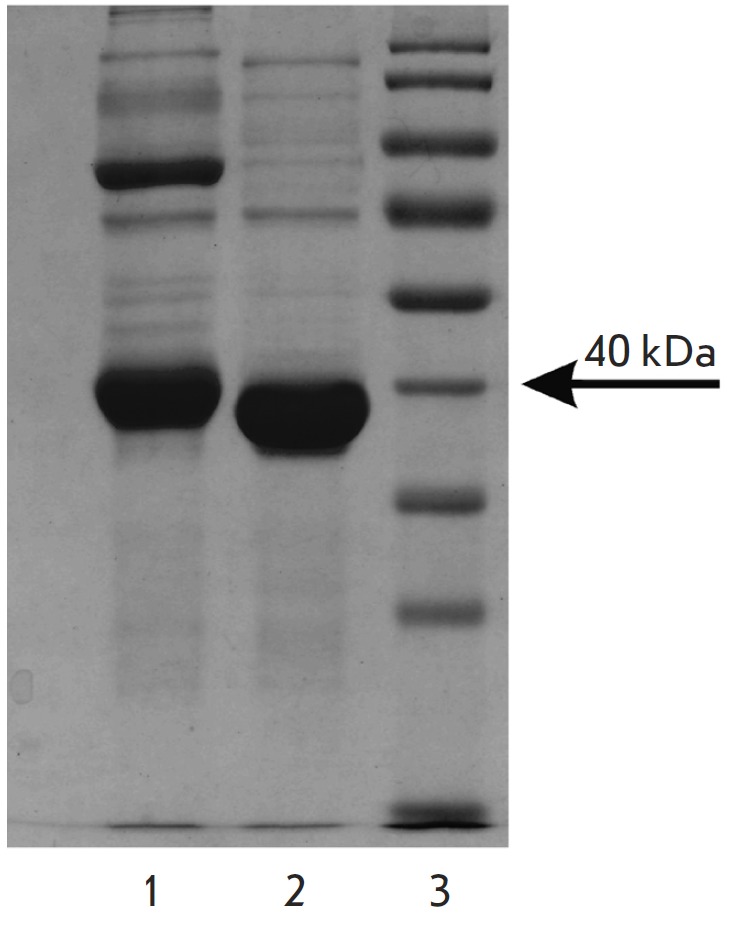
Electrophoresis of С. sporogenes MGL. 1 – enzyme with His-tag fragment, 2 – enzyme after thrombin detachment of Histag, 3 – standard molecular weight markers

The molecular weights of the native MGL preparations from the three sources mentioned above and* C. freundii *were determined by gel filtration. It was established that all the enzymes, independent of their source, are characterized by a tetrameric form. Figure 2 shows the data on the gel filtration of MGL from* C. sporogenes*. It was demonstrated that the molecular weights of the preparations from *C. sporogenes*, *C. tetani* and *P. gingivalis *after cleavage of the His-tag by thrombin are all equal approximately to 170 kDa, which corresponds to the tetrameric form. It should be noted that the oligomeric form was identified in the MGL preparation from *C. tetani *(characterized by the physiological activity of MGL) with a molecular weight of approximately 258 kDa. No dimeric forms were detected in the electrophoregrams of any of the preparations (*Fig. 3*), which excludes the aforementioned possibility that they form during the oxidation of the sulfhydryl groups of MGL under standard conditions of Laemmli electrophoresis. Therefore, the dimeric form of MGL is observed exclusively during the gel electrophoresis of denatured preparations from three sources containing a His-tag at the N-termini of polypeptide chains.

**Fig. 4:**
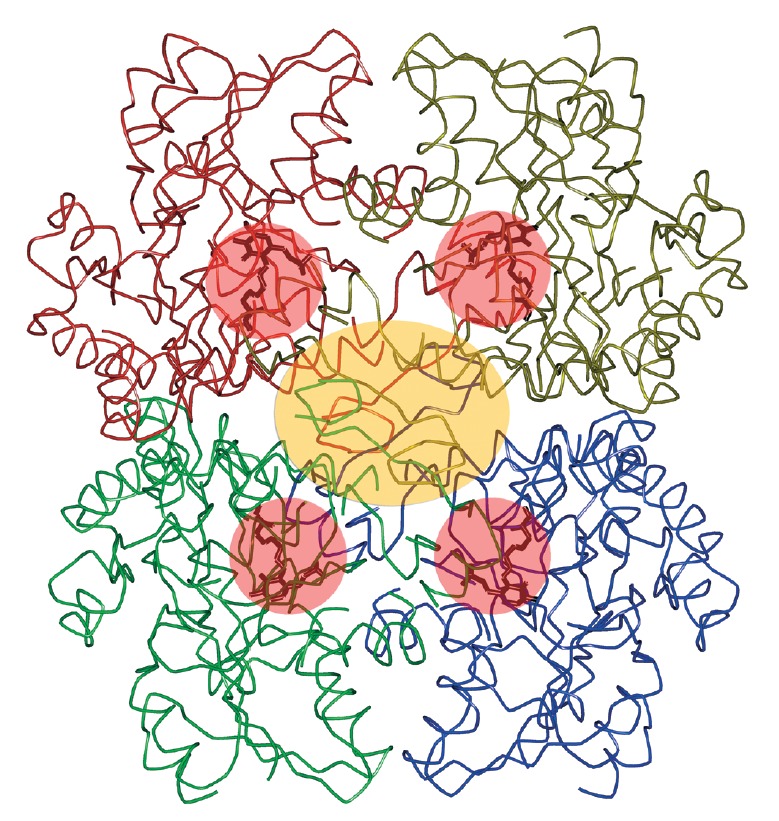
Tetramer of C. freundii MGL. The subunits are marked using different colors, active sites are shown in pink, and the contact region between catalytic dimers is shown in yellow

According to the X-ray diffraction analysis data, a tetrameric molecule of MGL from *C. freundii *consists of two catalytic dimers, each possessing two active sites formed by the residues of two subunits. The N-terminal domains of each subunit participate in the formation of a dimer, generating multiple hydrogen bonds with the residues of the C-terminal domain of the adjacent subunits and in the association of two catalytic dimers as they come into contact with the residues of the two C-terminal domains of the second catalytic dimer (Fig. 4) [26]. It is possible that the N-terminal His-tag present in the molecules of MGL from *C. sporogenes*, *C. tetani *and *P. gingivalis *forms additional bonds with residues of the C-terminal domain of the catalytic dimer and with the C-terminal residues of two subunits of the second catalytic dimer; thus, dimerization of the subunits can occur in denatured preparations.

The specific activity of the MGL preparations from* C. sporogenes*, *C. tetani*, and *P. gingivalis *determined after the cleavage of the His-tag by thrombin in the γ-elimination reaction of *L*-methionine turned out to be 1.5 times higher. The 50% increase in the specific activity of the preparations cannot be attributed exclusively to their minor additional purification after the treatment with thrombin. It is possible that the presence of a His-tag affects MGL activity.

In order to explain the variability in the catalytic efficiency of the enzymes and probable influence of the His-tag localized at the N-terminal regions of the polypeptide chains of the MGL from *C. sporogenes*,* C. tetani*, and *P. gingivalis *on enzyme activity, further investigations involving an X-ray diffraction analysis are required.

**Cytotoxicity of methionine γ-lyase from* C. freundii*, *C. sporogenes *and *C. tetani***

The calculated IC_50_ values for MGL of various origins on a panel of cell cultures are presented in Table 2. PC-3 prostate cancer and K562 human chronic erythroblastic leukemia cell cultures were the ones most sensitive to the action of the enzymes; their IC_50_ values were 0.1–0.4 and 0.4–1.3 U/ml, respectively. LnCap prostate cancer cells were the least sensitive: in the investigated concentration range, the IC_50_ value could not be determined for any of the enzymes. The sensitivity of K562 and MCF7 cells to the action of MGL is characterized by a significant variability.

**Table 2:** IC_50_ of MGL for several tumor cell cultures

Cell culture	IC_50_ of MGL, U/ml		
	С. freundii	C. sporogenes	C. tetani
K562	1.3	0.9	0.4
PC-3	0.1	0.4	-
LnCap	> 2.9	> 2.9	> 6.2
MCF7	0.5	0.04	3.2
L5178y	1.7	> 2.9	-
SCOV-3	-	-	5.3

The results obtained are indicative of the relatively high sensitivity of most of the cells that had been investigated to the action of MGL. Thus, the cytotoxicity of MGL is comparable to that of the other known enzymes, in particular, *L*-asparaginase from *E. coli*: with respect to the K562 and MCF7 cell cultures, the IC50 values for *L*-asparaginase from *E. coli *are equal to 0.8 and 10.9 U/ml, respectively [30]. The cytotoxicity level closest to that of MGL from *P. putida *was observed for the corresponding enzyme from *C. sporogenes*.

There is a statistically substantiated hypothesis regarding the direct dependence of the antitumor effect of the preparations whose effect is based on the destruction of another amino acid, *L*-asparagine (*L*Asparaginase) [31, 32]. In connection to this, the contribution of enzymatic activity to the materialiation of the cytotoxic effect of MGL is of considerable interest. A statistical analysis of the grouped data on the dependence of MGL cytotoxicity on *K*_m_ for various substrates revealed no relationships between these parameters. However, a tendency has been noted towards a positive relationship between *K*_m_ with respect to *L*-methionine and IC_50_ (r = 0.549, p = 0.100), which indirectly supports the existence of a relationship between enzymatic reduction of the methionine level in the medium and cytotoxic activity.

The identified tendency towards an increase in IC_50_ with increasing *K*_m_ may allow one to cautiously assume the probability of an increase in MGL cytotoxicity with increasing affinity to *L*-methionine. This does not contradict the existing concept regarding the enzymes used in oncology as medicinal products, whose antitumor effect is associated with increased sensitivity of cancer cells to the lack of amino acids.

## CONCLUSIONS

The determination of the kinetic parameters and cytotoxic activity of MGL from three bacterial sources demonstrated that the enzyme from *C. sporogenes *shows promise and requires further research. It is characterized by a minimal *K*_m_ value as compared to the other investigated enzymes and the highest cytotoxicity approaching that of MGL from *P. putida*.

The results obtained for the K562, MCF7, and PC-3 cell cultures allow one to consider further research into the *in vivo *antiproliferative activity of MGL and *in vitro *research into an extended panel of cell cultures as rather promising; there is a possibility of using the enzyme to design a novel antitumor agent.

## Abbreviations

DTT: dithiothreitol
His-tag: poly-histidine fragment
MGL: methionine γ-lyase
MSC: mesenchymal stromal cells
PLP: pyridoxal 5’-phosphate
